# Integrated metabolomic and transcriptomic analyses of *Dendrobium chrysotoxum* and *D. thyrsiflorum* reveal the biosynthetic pathway from gigantol to erianin

**DOI:** 10.3389/fpls.2024.1436560

**Published:** 2024-09-26

**Authors:** Lihang Xie, Qiuying Chen, Najing Cheng, Yue Zhang, Yao Ma, Yueteng Zhang, Kangdong Liu

**Affiliations:** ^1^ Tianjian Laboratory of Advanced Biomedical Sciences, Academy of Medical Sciences, Zhengzhou University, Zhengzhou, Henan, China; ^2^ School of Agricultural Sciences, Zhengzhou University, Zhengzhou, China; ^3^ Department of Pathophysiology, Basic Medicine Research Center, School of Basic Medical Sciences, Zhengzhou University, Zhengzhou, China

**Keywords:** dendrobium, erainin, biosynthetic pathway, metabolomics, transcriptomics

## Abstract

Erianin is one of the most representative bibenzyls with significant inhibitory activity against a wide range of tumor cells. However, the low erianin level in natural materials has severely inhibited its further development in health care. Our aim was to uncover the erianin biosynthetic pathway to lay the foundation for promoting its production. Firstly, we screened and obtained two *Dendrobium* species (*Dendrobium thyrsiflorum* stems with lower erianin content and *D. chrysotoxum* stems with higher erianin content), belonging to the same *Dendrobium* section (Chrysotoxae). A systematic analysis of bibenzyl structure and content in two stems revealed that gigantol might be an erianin biosynthetic intermediate, which was verified by introducing deuterium-labeled gigantol. Chemical structure analyses indicated that gigantol was modified by two kinds of enzymes (hydroxylases and O-methyltransferases), leading to erianin synthesis. Up-regulated hydroxylases and O-methyltransferases (OMTs) were screened out and were performed by molecular docking simulation experiments. We propose a rational biosynthetic pathway from gigantol to erianin, as well as relevant enzymes involved in the process. Our findings should significantly contribute to comprehensive resolution of the erianin biosynthetic pathway, promote its large-scale industrial production as well as contribute to biosynthesis studies of other bibenzyls.

## Introduction

Erianin (2-methoxy-5-[2-(3,4,5-trimethoxy-phenyl)-ethyl]-phenol), the representative bibenzyl compounds (Su et al., 2017), has been used in health care for the biological activities such as anti-inflammatory (Zhang et al., 2019a), anti-oxidant (Chen et al., 2019) and anti-bacterial (Yuan et al., 2018). Notably, it has been demonstrated to possess significant anti-tumor effects on various cancer cell lines *in vitro* and *in vivo* (Qiao et al., 2022). Erianin can inhibit the proliferation of different tumor cells through various mechanisms, inducing cell differentiation, apoptosis, or autophagy, arresting cell cycle, inhibiting tumor angiogenesis, and affecting cell migration and invasion (Zhang et al., 2019b). Overall, erianin is an active anti-cancer component with great potential. Erianin is mainly found in *Dendrobium chrysotoxum* (DC) stems, with only 0.12% of the yield, it can barely meet the needs of anti-cancer drug development and market needs (Luo et al., 2019; Qiao et al., 2022).

Artificially synthesized erianin can be a suitable strategy to compensate for its shortage of natural resources. Although the chemical synthesis of erianin has been previously reported, it has not been widely adopted because of high cost and the inevitable production of polluting by-products during its synthesis (Zou et al., 2008). Recent biosynthetic technological developments offer alternative opportunities for low cost erianin production. Naturally, the first step of synthetic biology is to elucidate biosynthetic pathway of target component. However, the biosynthetic pathway of erianin is not entirely clear (Adejobi et al., 2021).

Recently, the physiological and molecular mechanisms underlying bibenzyl compound biosynthesis were investigated using transcriptomic analyses of MethylJasmonate (MeJA)-treated *D. officinale* roots (Adejobi et al., 2021). Two candidate cytochrome P450 genes and nine other enzymatic genes might be functionally involved in bibenzyl biosynthesis. Generally, the first step in bibenzyl compound synthesis is the production of cinnamic acid catalyzed by phenylalanine ammonia-lyase (PAL) with L-Phenylalanine as substrate. Then, cinnamate 4-hydroxylase (C4H) catalyzes the conversion of cinnamic acid into two isomers (*m*-coumaric acid and *p*-coumaric acid). Finally, 3,3’5-trihydrobibenzyl is produced with subsequent catalyzation of CYP450, 4-coumarate-CoA ligase (4CL), and bibenzyl synthase (BBS), employing *m*-coumaric acid as substrate. However, the biosynthetic pathway between 3,3’5-trihydrobibenzyl and erianin is still unclear.

*D. thyrsiflorum* (DT), belonging to the same *Dendrobium* section (Chrysotoxae) as DC, was found to contain smaller amounts of erianin (Luo et al., 2019). In this study, a combined metabolome and transcriptome approach was employed to investigate erianin biosynthesis differences between DT and DC, shedding light on how erianin is synthesized in *Dendrobium* plants. Gigantol was found to be a possible intermediate in the biosynthesis pathway of erianin, and the structural genes related to the synthesis of erianin were initially identified. Our studies are of great significance for comprehensively elucidating erianin biosynthetic pathway and would provide a guidance for future synthetic biology strategies.

## Materials and methods

### Plant materials

Two-year-old DC and DT stems were collected from the same plantation at Ning ‘er Hani Yi Autonomous County, Pu ‘er City, Yunnan, China (101°03’8.79”N, 23°03’16.52”E). Each stem was divided into two parts along growth direction. One-half of the stem was used for metabolomic analysis, and the other half was used for transcriptomic analysis. Three biological replicates were performed for each sample. All samples were immediately frozen in liquid nitrogen and kept at -80° for further use.

### Widely targeted metabolome analysis

Widely targeted metabolome analysis was performed by Metware Biotechnology Co., Ltd. (Wuhan, China) following their standard procedures. Briefly, 100 mg of lyophilized powder was dissolved with 1.2 mL 70% methanol solution, vortexed 30 seconds every 30 minutes for 6 times in total, and then placed in a refrigerator at 4°C overnight. After centrifugated at 12000 rpm for 10 min, the extracts were filtrated through a 0.22 μm polytetrafluoroethylene (PTFE) membrane (MF, Millipore) before UPLC-MS/MS analysis.

The sample extracts were analyzed using an UPLC-ESI-MS/MS system (UPLC, SHIMADZU Nexera X2; MS, Applied Biosystems 4500 Q TRAP). The analytical conditions were as follows, UPLC: column, Agilent SB-C18 (1.8 µm, 2.1 mm x 100 mm); The mobile phase was consisted of solvent A, pure water with 0.1% formic acid, and solvent B, acetonitrile with 0.1% formic acid. Sample measurements were performed with a gradient program that employed the starting conditions of 95% A, 5% B. Within 9 min, a linear gradient to 5% A, 95% B was programmed, and a composition of 5% A, 95% B was kept for 1 min. Subsequently, a composition of 95% A, 5.0% B was adjusted within 1.1 min and kept for 2.9 min. The flow velocity was set as 0.35 mL per minute; The column oven was set to 40°C; The injection volume was 4 μL. The effluent was alternatively connected to an ESI-triple quadrupole-linear ion trap (QTRAP)-MS.

Metabolite identification and quantification were performed using the self-built MetWare database and other public databases. The scheduled multiple reaction monitoring (MRM) method was previously described by Chen et al (Chen et al., 2013). All the bibenzyl were further screened out based on the chemical structures.

### Introduction of label and identification

The introduction of ^2^H (D) label was performed by adding D-gigantol dissolved with DMSO, which was chemically synthesized in our laboratory and verified by NMR, at a final concentration of 50 mM to the buffer solution (10 mM MES + KOH, pH 6.5). The stems pieces of DC from six independent plants were mixed and incubated for 5 h in buffer solution under vacuum. At the end of the incubation, stems were snap-frozen in liquid nitrogen. They were subsequently extracted using 100% methanol and filtered through a 0.22 μm filter membrane.

Chromatographic analysis was carried out using a Waters Acquity UPLC system (Waters Corp., Milford, MA, USA) with a waters BEH C18 column (1.7 μm, 2.1 × 50 mm) at a temperature of 40°C. Gradient elution with 0.1% of formic acid aqueous solution (A) and acetonitrile solution (B) was employed at a flow rate of 0.4 mL/min, with the following gradient program: 0-1 min 5% B, 1–7 min 5%–98% B, 7–10 min 98% B. The injection volume was 1 μL.

Mass spectrometric detection was performed on Waters Xevo G2-XS QTOF mass spectrometer (Waters Corp., Milford, MA, USA) equipped with an ESI source. Source conditions included a source temperature and desolvation gas temperature of 110 and 400°C, respectively, cone gas flow and desolvation gas (N_2_) flow of 100 and 800 L/h, respectively, and sampling cone voltage of 40 V, with a capillary voltage of 2.5 kV for positive ion mode. TOF parameters included a full scan analysis with a mass range from 50 to 1200 Da and collision energy set as 6.0 eV for low-energy scan and 20–30 eV for high-energy scan.

### RNA extraction, illumine sequencing, and transcriptome data analysis

Six total RNA samples (two different groups with three replicates) were isolated using the RNAprep Pure Plant Plus Kit (#DP441; Tiangen, http://www.tiangen.com) according to the manufacturer’s instructions, and a 1% agarose gel and a Nano spectrophotometer (IMPLEN, CA, USA) were used to test the concentration of the total RNA. The cDNA library construction and RNA-seq were performed by Metware Biotechnology Co., Ltd. (Wuhan, China). A total of 1 μg RNA per sample was used to generate a cDNA library using the NEBNext^®^ UltraTM RNA Library Prep Kit (NEB, USA) according to the manufacturer’s instructions. The libraries were then sequenced on an illumina Hiseq platform. The original data were filtered to remove reads with adapters using fastp v 0.19.3. Then, the clean reads were mapped to the *D. catenatum* reference genome (https://www.ncbi.nlm.nih.gov/datasets/genome/GCF_001605985.2/) using the default parameters of Hisat2 software (Li et al., 2020). Fragments per kilobase of transcript per million mapped reads (FPKM) were used for transcription or quantification of gene expression levels. An absolute log 2 (fold change) ≥1 and false discovery rate (FDR) < 0.05 were used as thresholds for the identification of differentially expressed genes (DEGs) using DESeq2 software. Gene Ontology (GO) enrichment analysis was performed using the GOSeq R package (corrected *p*-value < 0.05). Pathway analysis was performed to elucidate the significant pathways of DEGs using Kyoto Encyclopedia of Gene and Genomes (KEGG) (http://www.genome.jp/kegg).

### Phylogenetic and molecular evolutionary analyses

The Hydroxylases and OMTs from different species were selected from the National Center for Biotechnology Information (NCBI) database (https://www.ncbi.nlm.nih.gov/) for the construction of a maximum likelihood phylogenetic tree. Multiple sequence alignment was performed with DNAMAN version 5.2.2. Molecular Evolutionary Genetics Analysis (MEGA) version X was applied for the phylogenetic and molecular evolutionary analysis. The phylogenetic tree was annotated using Evolview v3 (Zhang et al., 2012).

### Homologous modelling and molecular docking

The homology structural models of hydroxylases and OMTs were built by SWISS-MODEL modelling server. The model quality was validated via ProSA-web (Wiederstein and Sippl, 2007), QMEAN version 4.3.0 (Benkert et al., 2008) and SAVES v6.0 (https://saves.mbi.ucla.edu/). The 3D structures of gigantol (PubChem CID: 3085362) and Compound 1 (PubChem CID: 153349) were obtained in structural data format (SDF) from PubChem database (https://pubchem.ncbi.nlm.nih.gov/), while that of Compound 2 was prepared using Chem3D by CambridgeSoft. The differentially expressed Hydroxylases and OMTs from DC were subjected to an In-silico molecular docking study with the help of AutoDockTool-4.2 (Morris et al., 2009), in which the grid box was set to X = 108.15, Y = 108.15 and Z = 108.15 with spacing = 0.858 Å for Compound 1, X = 92.05, Y = 92.05 and Z = 92.05 with spacing = 0.731 Å for gigantol (3) and Compound 2, covering all the reported active residues. These tools also aid to forecast the binding stability between the two molecules by means of the scoring function. Individually the protein-ligand was observed through the Pymol (Yuan et al., 2017) and Discovery Studio 2019 Client (An et al., 2020).

### Statistical analysis

The experimental data, consisting of three independent biological replicates, were subjected to statistical analysis using SPSS version 21.0. One-way analysis of variance was followed by Duncan’s tests, with a significance level set at *p* < 0.05. A heatmap of erianin biosynthesis-related genes was constructed based on transcriptome data using TBtools. The evolution tree of differentially expressed hydroxylases and OMTs was performed by OmicShare.

## Results and discussion

### Selection of materials for investigating erianin biosynthetic pathway

**Figure 1:**
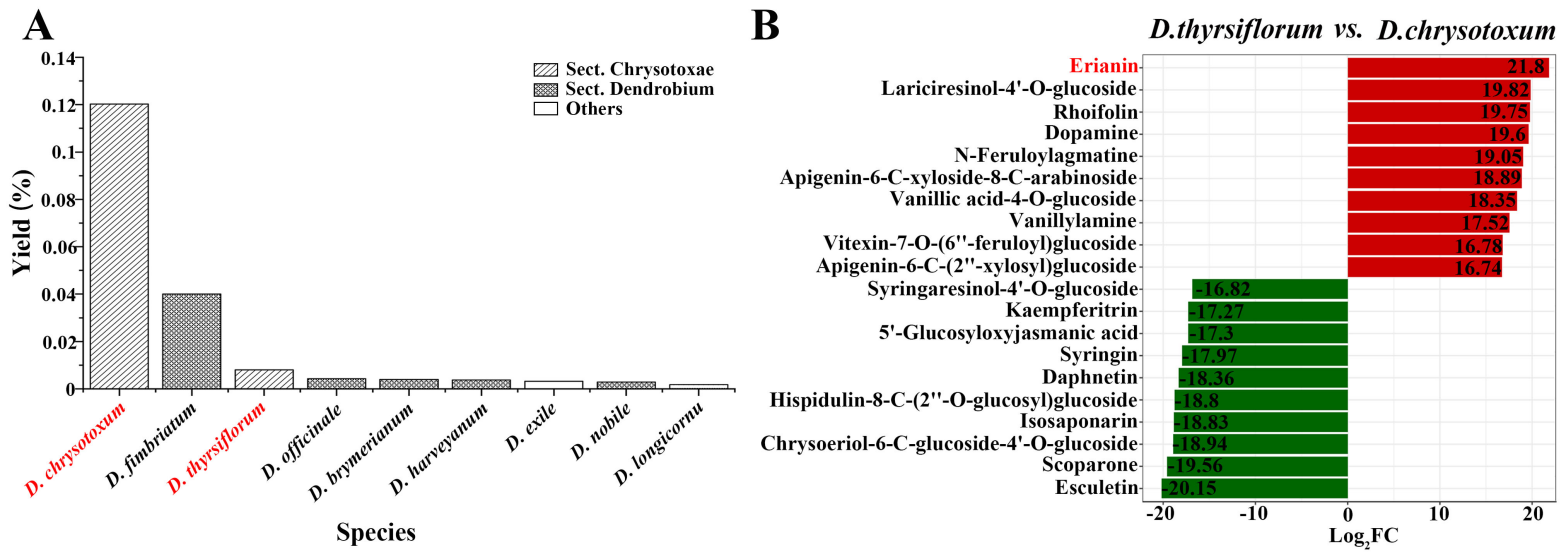
Erianin content in different wild *Dendrobium* species **(A)**; Top 10 differential secondary metabolites in DT and DC stems **(B)**.

Only a few wild *Dendrobium* species were found to contain erianin, among which DC had the highest erianin content, accounting for about 0.12% of the mass fraction (Luo et al., 2019). Although DT and DC belong to the same *Dendrobium* section, the content of erianin in DT stem was only 0.008% (**Figure 1A**). Moreover, comparative metabolomic analysis showed that erianin was the secondary metabolite that differed the most in content between the two *Dendrobium* species (**Figure 1B**, **Supplementary Table S1**). Therefore, DC and DT are good research materials for exploring the biosynthetic pathway of erianin.

### Gigantol is a possible intermediate in erianin biosynthesis pathway

**Figure 2:**
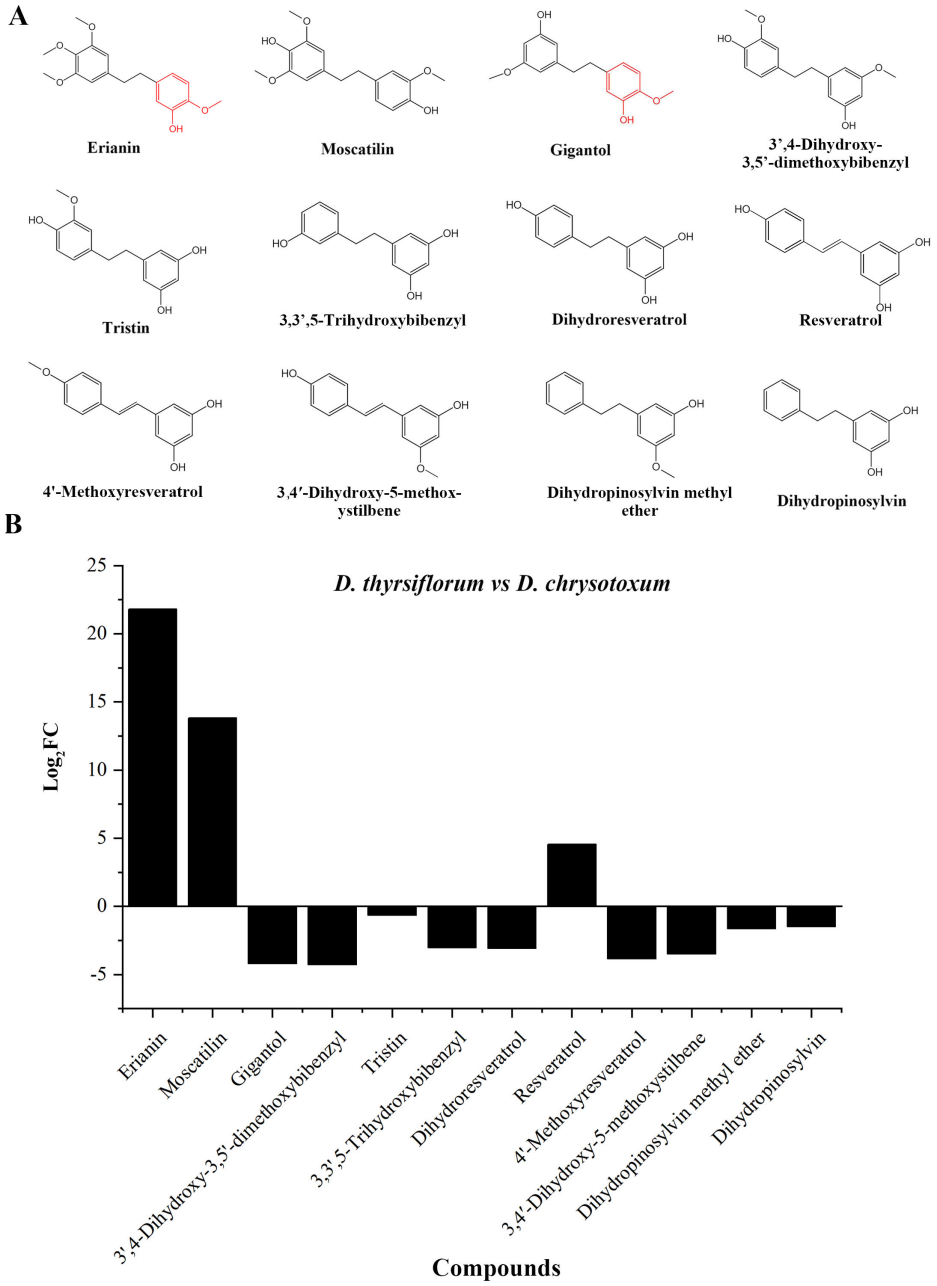
Bibenzyl compounds identified in the metabolome. **(A)** Structure of identified bibenzyl compounds; **(B)** Difference in the relative content of identified compounds in the stems of DT and DC.

To discover possible intermediates in the erianin synthesis pathway, the substances detected by the metabolome were further screened and analyzed. A total of 11 bibenzyl compounds structurally similar to erianin were discovered (**Figure 2**). Based on chemical structure analysis, gigantol and erianin were the most structurally similar, especially in one of the benzene rings, where both were methoxy in the para position and hydroxyl in the interposition. Moreover, the content of erianin was significantly higher than that of gigantol in DC, when compared to DT. Similar results also have been reported previously (Duo et al., 2018). Therefore, gigantol is more likely to be consumed and further modified to synthesize erianin, resulting in low content of gigantol in DC stems.

### Validation of gigantol as a potential intermediate for erianin biosynthesis

**Figure 3:**
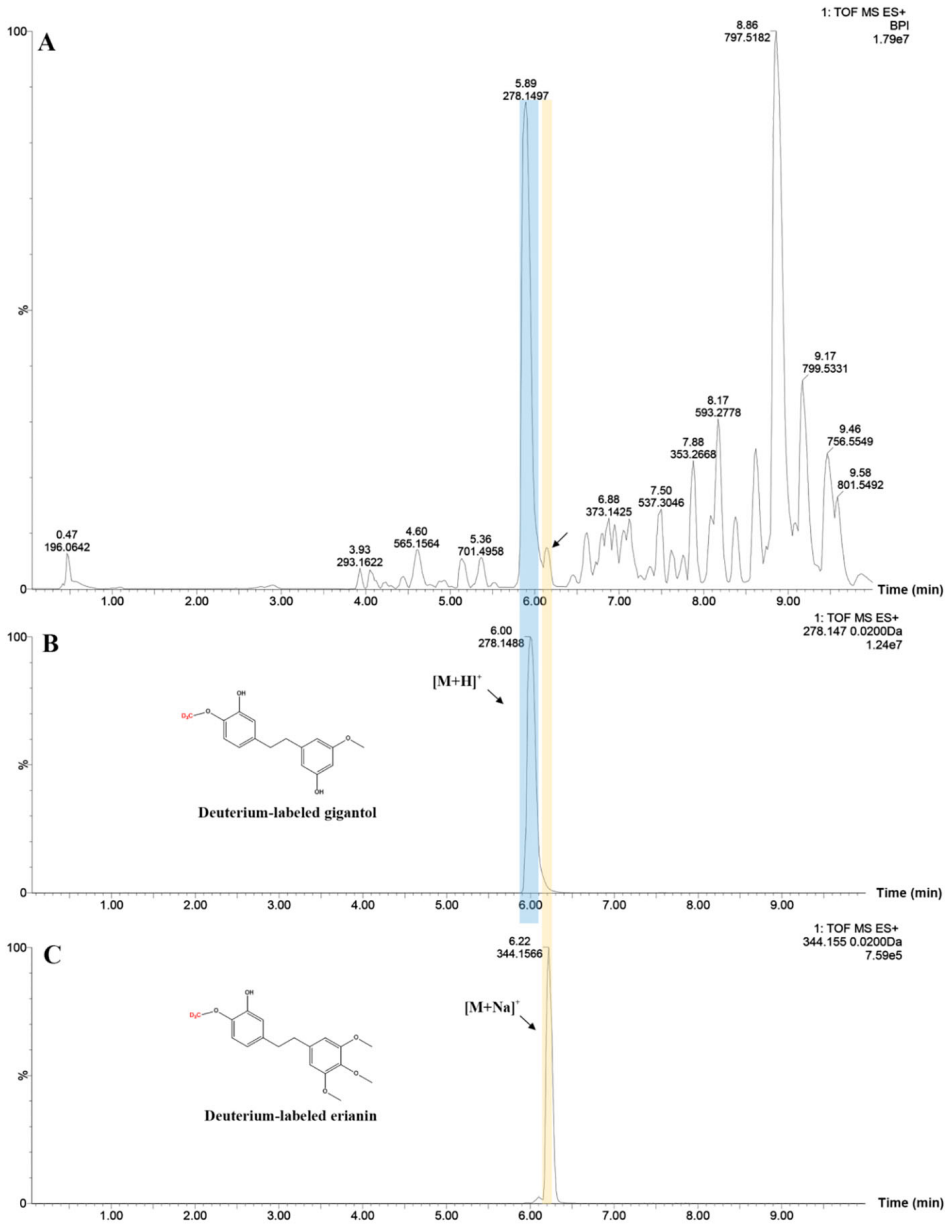
Chromatographic profiles of treated DC stems **(A)**, deuterium-labeled gigantol **(B)** and erianin **(C)**.

To determine if gigantol could act as an intermediate substance for erianin biosynthesis, the deuterium-labeled gigantol was employed. By vacuuming, deuterium-labeled gigantol was pumped into DC stems, which were previously cut into small pieces. LC-MS results showed that deuterium-labeled gigantol and erianin were detected in treated DC stems extracts (**Figure 3**, **Supplementary Figure S1**). Indeed, isotopic tracers are used to both trace metabolic pathways and quantify fluxes through these pathways (Schwender, 2008; Chassy et al., 2014). But, the use of deuterium feeding in the way is quite rare in the studies on the biosynthetic pathway of plant secondary metabolites, except the study using mechanism-directed deuterium labeling for the tracing of benzylisoquinoline alkaloid biosynthesis in *Papaver somniferum* (Vavricka et al., 2022). The deuterium-labeled gigantol and erianin at the same site were designed and produced by our laboratories based on their chemical structures, which provided an excellent tool for the validation of previous inferences. Since deuterium is not a common element in nature, the detection of deuterium-labeled erianin in DC stems after being fed with deuterium-labeled gigantol further suggests that gigantol is likely to be one of the potential intermediates for erianin biosynthesis. In addition to this, trace amounts of erianin were detected possibly due to isotope discrimination, which incorporates D relative to the equivalent H during reactions, and the inactivation of enzymes in stems after isolation (Werner and Schmidt, 2002).

### Overview of the transcriptome sequencing

In order to investigate the molecular mechanism of erianin biosynthesis, RNA-Seq was employed for transcriptome analysis. Stems of DT and DC were collected and used to construct six sequencing libraries, namely DC1, DC2, DC3, DT1, DT2, and DT3. In total, 40.14 Gb of raw data with 94.04% of average clean Q30 were obtained from the six libraries (**Supplementary Table S2**). Of all clean reads, 72.07 to 80.80% were mapped to the *D. catenatum* reference genome. A total of 29,028 expressed genes in DC stems and 27,842 expressed genes in DT stems were detected. The correlation analysis and principal component analysis (PCA) of samples showed high reproducibility between the biological replicates (**Supplementary Figure S2**).

### DEG identification and enrichment analysis

**Figure 4:**
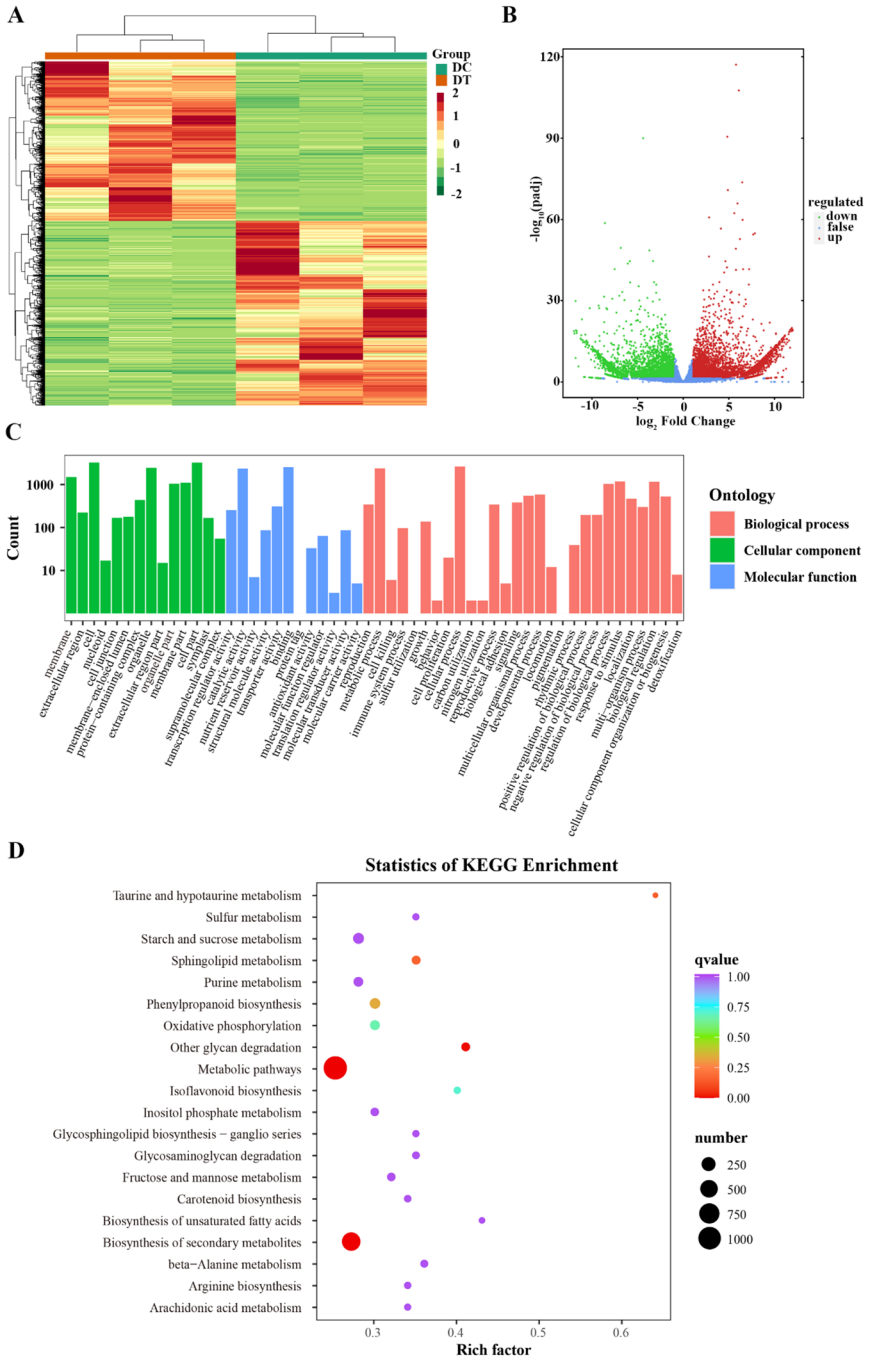
Analysis of differentially expressed genes (DEGs) between DC and DT. **(A)** Heat map of DEGs based on hierarchical clustering analysis; **(B)** Volcano plots of DEGs. The red dots represent the significantly upregulated genes, and the green dots represent the significantly downregulated genes; **(C)** GO enrichment analysis of DEGs in the three functional groups; **(D)** Scatter plots of top 20 KEGG pathways with the most significant enrichment.

A total of 7,992 DEGs were identified, including 3,706 downregulated, and 4,286 upregulated genes, in DC relative to DT comparisons with fold change ≥ 2 and FDR (False Discovery Rate) < 0.05 as filter criteria (**Figures 4A, B**, **Supplementary Table S3**). Enrichment analysis of GO functions showed that all DEGs could be divided into three functional groups: biological processes, cellular components, and molecular functions (**Figure 4C**). As expected, many DEGs were associated with metabolic and cellular processes in the “biological process” category. In the cellular component class, numerous genes were annotated to participate in “cell part”, “cell”, and “organelle” processes. In the molecular function category, the most DEGs were enriched in the ‘binding’ and ‘catalytic activity’ subcategories. The KEGG pathway enrichment analysis revealed that DEGs were mainly enriched in ‘metabolic pathway (ko01100)’ and ‘biosynthesis of secondary metabolites (ko01110)’ (**Figure 4D**).

### The differences in erianin biosynthesis pathway between DT and DC

**Figure 5:**
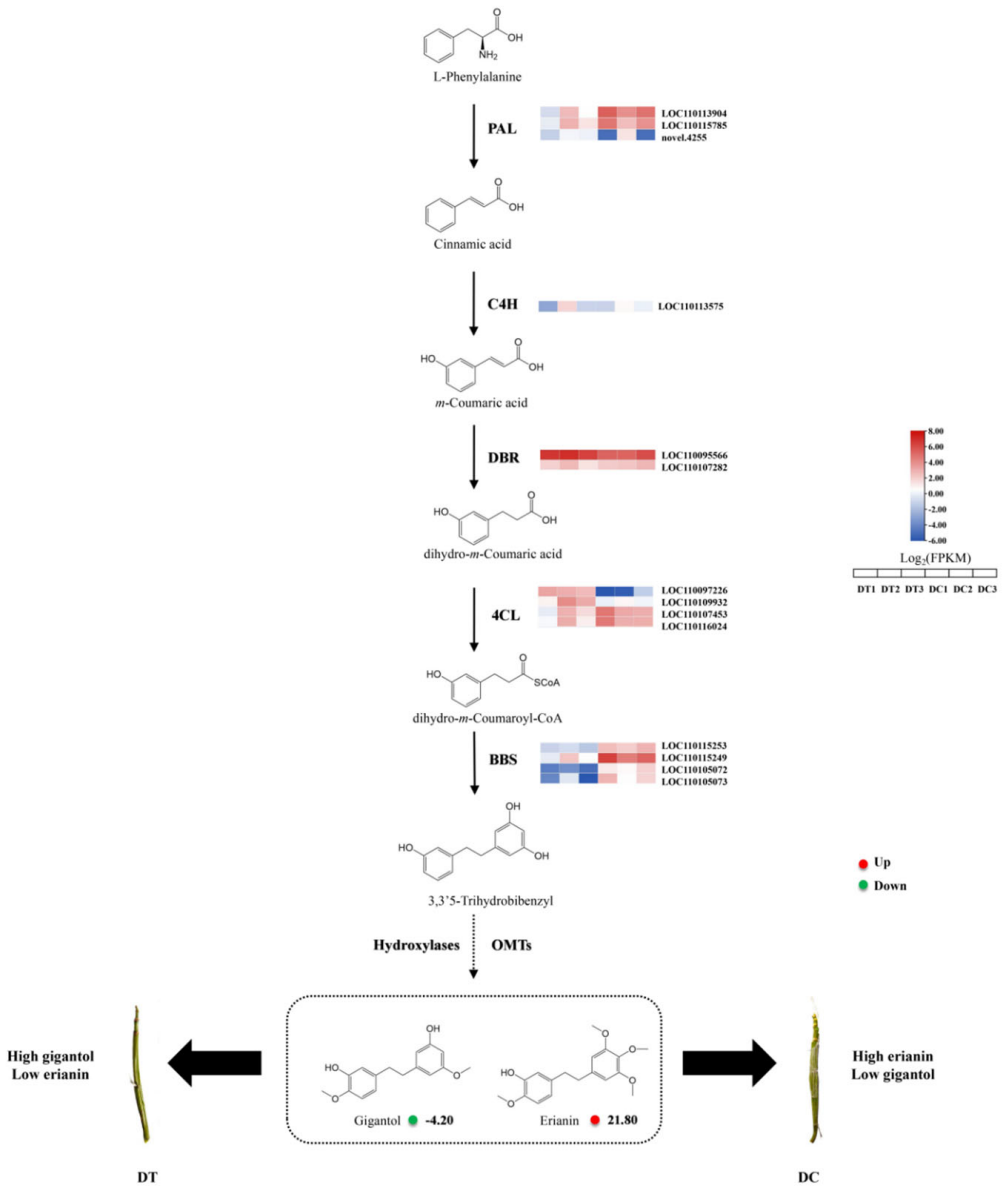
Pathways and genes involved in the biosynthesis of erianin in DT and DC. The pathway was built using published literatures and KEGG pathway analysis. The colored grids on the top or right of each gene is the expression heat map of key enzyme genes for the biosynthesis of erianin between DT and DC. The red solid circles represent Log_2_ fold change (Log_2_FC) of metabolites (DT *vs* DC). PAL, phenylalanine ammonia lyase; C4H, cinnamate 4-hydroxylase; 4CL, 4-coumadin CoA ligase; BBS, bibenzyl synthase (or bibenzyl synthase-like); DBR, double bond reductase; OMTs, O-methyltransferases.

To understand the biosynthesis mechanism of erianin, the integrative metabolomic and transcriptomic analysis between DT and DC was carried out (**Figure 5**). The expression of four BBS genes (LOC110115253, LOC110115249, LOC110105072, and LOC110105073) were significantly higher in DC than that in DT stems. The high expression of BBS genes leads to sufficient accumulation of intermediates for the synthesis of erianin in DC stems. In orchid species, BBS was first isolated and purified from *Bletilla striata*, and bibenzyls were formed in large amounts with the activity of BBS in orchid rhizomes (Reinecke and Kindl, 1994). Therefore, BBS genes played a key role in bibenzyl biosynthesis in Orchid species. Similar conclusions also were found in *D. sinense* BBS. *DsBBS* genes were characterized by the combination of genes expressions and bibenzyl contents, and the key *DsBBS* gene was identified to specifically catalyze the cyclization and aromatization of 4-coumaryol-CoA and malonyl-CoA to generate resveratrol (Chen et al., 2022). Moreover, the content of resveratrol in DC stems was significantly higher than its content in DT, which was consistent with the results of the transcriptome (**Supplementary Table S1**). Conversely, resveratrol is catalyzed to produce more 4’-Methoxyresveratrol and 3,4′-Dihydroxy-5-methoxystilbene by OMTs in DT stems. Although the amount of dihydroresveratrol in DC stems was slightly less than in DT, the difference was statistically insignificant. Furthermore, dihydroresveratrol is catalyzed by the combined action of OMTs and hydroxylases to produce gigantol and erianin. Similar results were also found in *D*. *officinale*, where 3,3’5-trihydrobibenzyl, the isomer of dihydroresveratrol, is regarded as the synthetic intermediates of gigantol and erianin with the catalyzation of OMTs and hydroxylases (Adejobi et al., 2021). Therefore, differences in the expression of OMT and hydroxylases are likely to be the main factors responsible for differences in the contents of gigantol and erianin in DT and DC.

### Candidate hydroxylases and OMTs related to erianin biosynthesis

**Figure 6:**
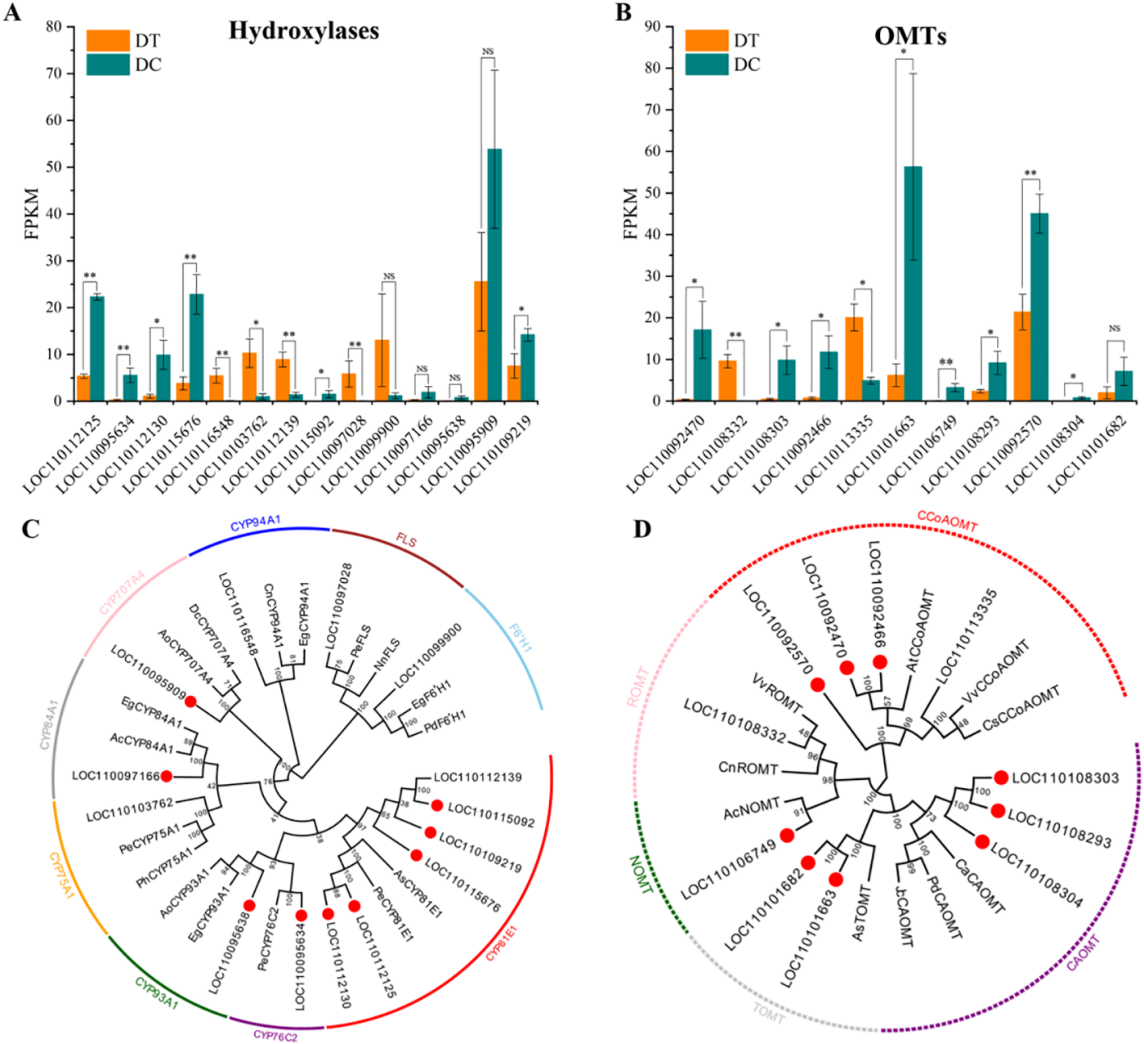
The comprehensive analysis of differentially expressed hydroxylases and OMTs. All the differentially expressed hydroxylases **(A)**, and OMTs **(B)** between DT and DC. *, 0.01 < *P* ≤ 0.05; **, *P* ≤ 0.01; NS, not significant. Phylogenetic analysis of hydroxylases **(C)** and OMTs **(D)**. Numbers on branches indicate the bootstrap percentage values calculated from 1000 bootstrap replicates. Genes marked with red circles show that it was significantly up-regulated in DC stems (Log2FC>1, p<0.05). Sequence accession numbers are given in **Supplementary Table S4**.

To further identify key genes that may play an important role in the biosynthetic pathway of erianin, the differentially expressed hydroxylases and OMTs were analyzed (**Figures 6A, B**). A total of 14 hydroxylases and 11 OMTs with differential expression were identified based on the comparative transcriptome analysis. Among them, the expression of 9 hydroxylases and 9 OMTs were significantly upregulated and positively correlated with the accumulation of erianin in DC stems.

A neighbor-joining phylogenetic analysis was conducted using the selected hydroxylases and OMTs in this study, and those previously reported in other plants by MEGA X. Hydroxylases were divided into nine groups with the most belonging to cytochrome P450 (CYPs) family (**Figure 6C**). CYPs are monooxygenases encoded by a family of super genes, which are involved in a variety of plant metabolic reactions playing important roles in plant growth and stress response regulation mechanisms (Cui et al., 2020). Five upregulated hydroxylases in DC stems belong to the CYP81E1 group. CYP81E1 proteins, also known as isoflavone 2’-hydroxylase-like, share a high sequence homology with *Apostasia shenzhenica* and *Phalaenopsis equestris*, which have a close divergence time with *D*. *catenatum* (Zhang et al., 2020). Isoflavone 2’-hydroxylase also has been identified in soybean (Overkamp et al., 2000), chickpea, and alfalfa (Liu et al., 2003), and recombinant CYP81E1 were reported to catalyze 2’-hydroxylation of formononetin (7-hydroxy, 4’-methoxyisoflavone) and the 2’- and 3’-hydroxylation of daidzein (7,4’-dihydroxyisoflavone) *in vitro* in yeast microsomes (Akashi et al., 1998).

According to the phylogenetic analysis, differentially expressed OMTs were divided into 5 groups, including caffeoyl-CoA O-methyltransferase (CCoAOMT), trans-resveratrol di-O-methyltransferase (ROMT), (RS)-norcoclaurine 6-O-methyltransferase (NOMT), tricetin 3’,4’,5’-O-trimethyltransferase (TOMT), and caffeic acid 3-O-methyltransferase (CAOMT) (**Figure 6D**). The up-regulated OMTs mainly belong to CCoAOMT and CAOMT groups. Based on protein’s size, substrate preference, and Mg^2+^ dependence, plant OMTs could be categorized into two major classes (Noel et al., 2003; Lam et al., 2007). Class I OMTs, also called CCoAOMTs, vary from 231 to 248 amino acids and require Mg^2+^ to perform its methylation. Unlike Class I OMTs, Class II OMTs are larger in length (344-383 aa) and act on a diverse group of compounds, such as CAOMT, ROMT (resveratrol O-methyltransferase), and others. Both CCoAOMT and CAOMT belong to the family of S-adenosyl-L-methionine (SAM) dependent O-methyltransferase and catalyze the conversion of caffeoyl-CoA to feruloyl-CoA, and caffeic acid into ferulic acid in lignin biosynthesis, respectively (Zhang et al., 2015; Narnoliya et al., 2021). CCoAOMTs and CAOMTs have been identified in a variety of vascular plants, including poplar (Zhong et al., 2000), alfalfa (*Medicago sativa*) (Marita et al., 2003), tobacco (*Nicotiana tabacum*) (Zhong et al., 1998), Arabidopsis (*A. thaliana*) (Do et al., 2007), and maize (*Zea mays*) (Yang et al., 2017). Lignin is the main component of *Dendrobium* stems (Yang and Liu, 2014; Mantovani et al., 2018), so the identification of CCoAOMTs and CAOMTs in DT and DC is reasonable. Moreover, CCoAOMTs and CAOMTs were found to be possibly redundant with respect to the methylation (Inoue et al., 1998). Therefore, the discovery of up-regulated CCoAOMTs and CAOMTs in DC stems also suggests that the biosynthesis of erianin could potentially occur via two parallel pathways.

### Molecular docking analysis and a proposed biosynthetic pathway from gigantol to erianin

Based on the above findings, a biosynthetic pathway from gigantol to erianin was proposed. Theoretically, the biosynthesis of erianin from gigantol normally required the modifications by hydroxylases and OMTs. However, it was found that gigantol would generate unknown compounds, whose chemical structure was not recorded in the databases (SciFinder and PubChem), when not first modified by hydroxylases (Bari et al., 2021). In contrast, gigantol is more likely to be first modified by OMTs to produce compound 1 (5-(2-(3,5-dimethoxyphenyl)ethyl)-2-methoxy-, 71135-71-2), then by hydroxylases to produce compound 2 (4-[2-(3-hydroxy-4-methoxyphenyl)ethyl]-2,6-dimethoxy-, 833826-33-8), which is an isomer of moscatilin (a common secondary metabolite in DC stems) (Xia et al., 2023), and finally by OMTs to produce erianin. Therefore, the proposed biosynthetic pathway of gigantol firstly modified by OMTs is relatively reasonable.

**Figure 7:**
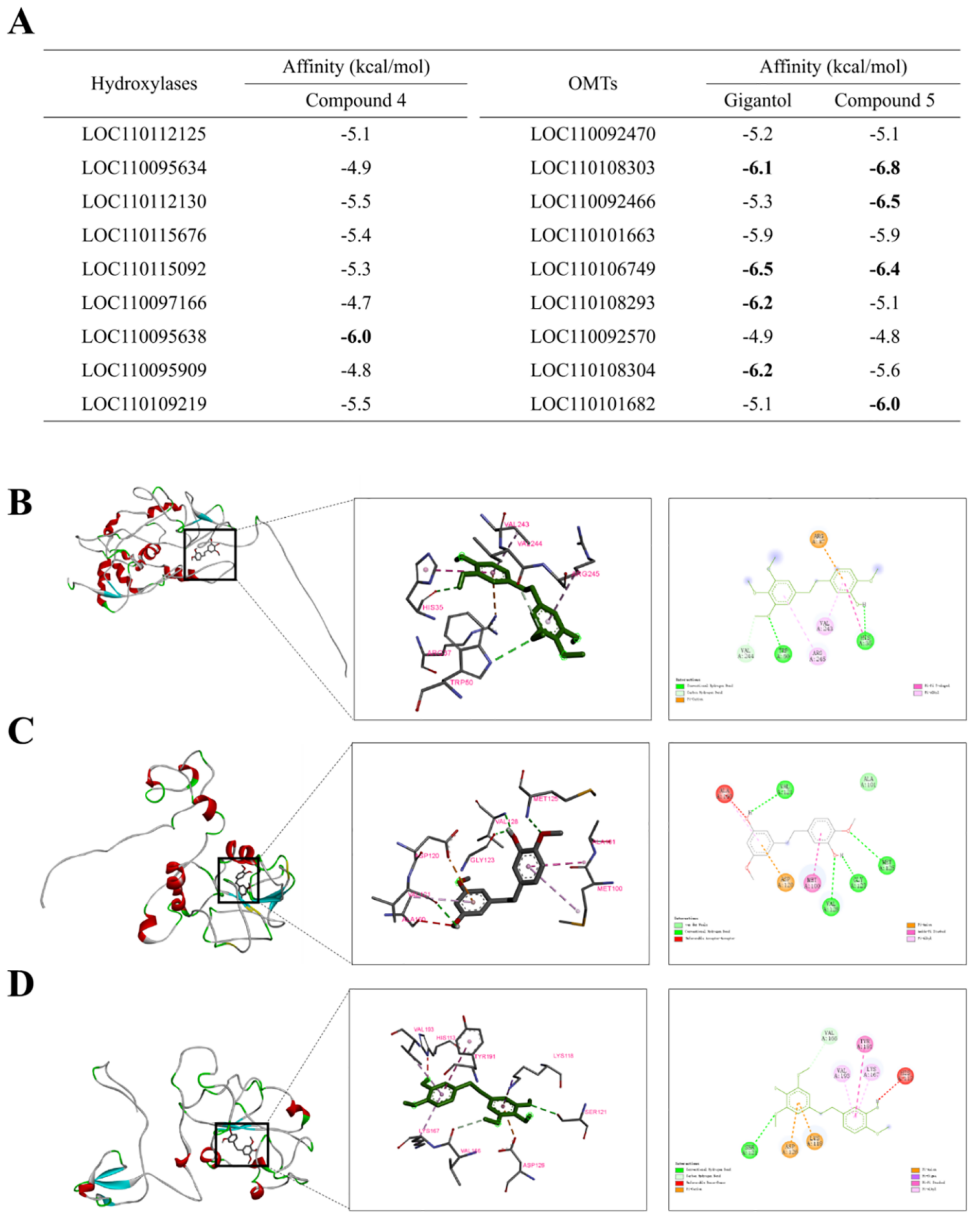
Results of molecular docking between substrates (compound 1, gigantol and compound 2) and key enzymes. **(A)** binding energy between the individual compounds and target proteins predicted by AutoDock Vina. The global diagrams and the details and the 2D diagrams of the docking between compound 1 and LOC110095638 **(B)**, gigantol and LOC110106749 **(C)**, compound 2 and LOC110108303 **(D)**.

To further refine the proposed biosynthetic pathway from gigantol to erianin, the possible substrates of the selected hydroxylases and OMTs were further analyzed using molecular docking. The three-dimensional protein model of hydroxylases and OMTs, which is positively correlated with the accumulation of erianin, was constructed using SWISS-MODEL and evaluated using UCLA-DOE LAB server v6.0 (Colovos and Yeates, 1993). Then, gigantol and compound 2 were docked into the active sites of OMTs as the substrate, while compound 1 was docked into that of hydroxylases. Most of the binding affinity predicted by AutoDock Vina were less than -5.0 kcal/mol, suggesting that they were relatively stable (**Figure 7A**). According to the standard of binding energy less than -6.0 kcal/mol, one tightly bound hydroxylase for compound 1 was screened out, while four OMTs were screened out in both gigantol and compound 2, respectively. These intermolecular interaction residues are listed in **Supplementary Table S5**. For compound 1, gigantol, and compound 2, the conformation with the smallest binding energy is shown in **Figure 7**. Protein LOC110095638 had the best docking score on interaction with compound 1. Two hydrogen bonds (TRP 50 and HIS 35) and two Pi-Pi interactions (VAL 243 and AGR 245) were found between Protein LOC110095638 and compound 1 (**Figure 7B**). Conversely, four hydrogen bonds (VAL 121, VAL 128, GLY 123, and MET 125) were found between protein LOC110106749 and gigantol, which also resulted in stronger combination ability and lower affinity (**Figure 7C**). More Pi interaction, including Pi-Cation, Pi-Anion, Pi-Pi and Pi-Alkyl, were found between protein LOC110108303 and compound 2 (**Figure 7D**).

**Figure 8:**
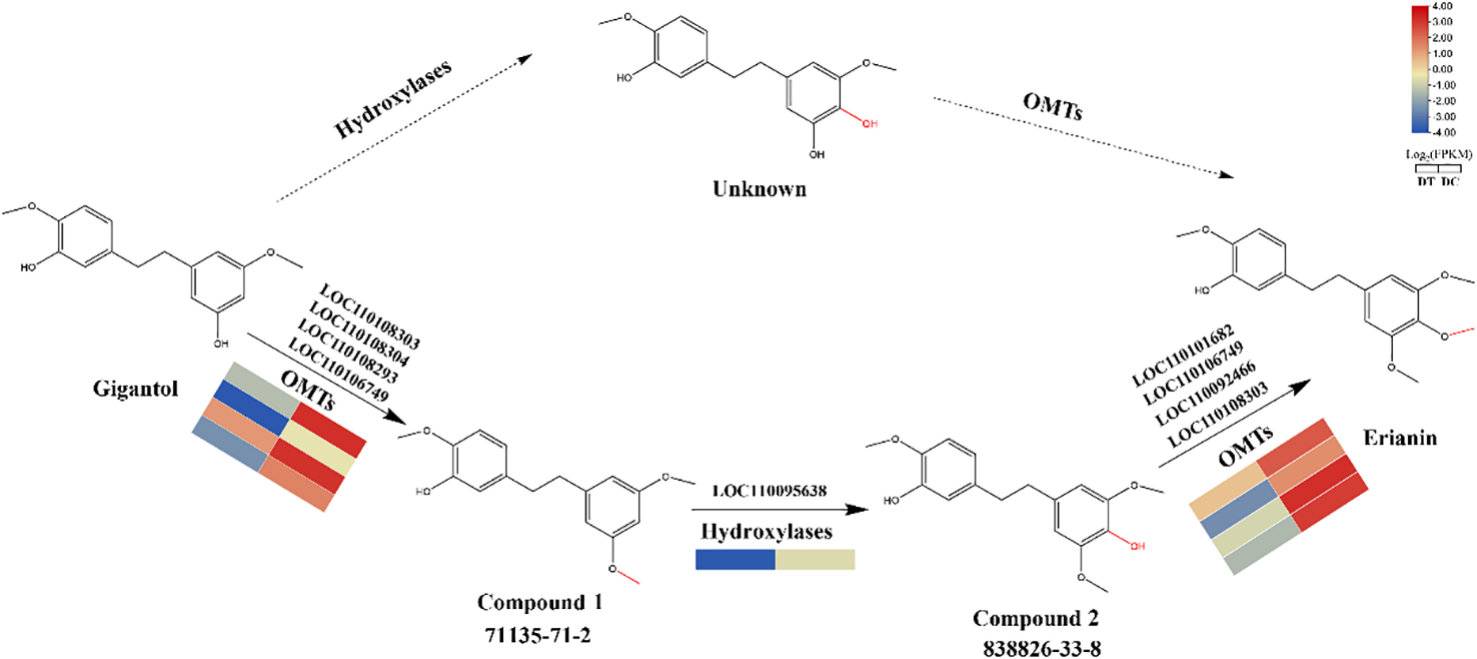
Hypothesized biosynthetic pathway from gigantol to erianin. Solid lines represent more likely biosynthetic pathways. Candidate genes were predicted according to molecular docking results.

Overall, molecular docking results revealed the possible substrates of identified hydroxylases and OMTs and their catalytic mechanism. Moreover, potential key catalytic enzymes were further predicted in each step of the biosynthetic pathway from gigantol to erianin (**Figure 8**).

## Conclusions

After a systematic analysis of benzyl compounds in DT and DC stems with significant differences in the content of erianin, gigantol was found to have similar structures with erianin and negatively correlated in content. Furthermore, deuterium-labeled gigantol was innovatively used to feed DC stems, and deuterium-labeled erianin was detected, suggesting gigantol might be one of the potential biosynthetic intermediates of erianin. To explore the biosynthetic pathway from gigantol to erianin in depth, comprehensive comparative metabolomic and transcriptomic analyses of DT and DC were conducted. The potential differentially expressed hydroxylases and OMTs that may play a key role in the pathway were screened out. The biosynthetic pathway from gigantol to erianin was proposed and the key enzymes during the biosynthesis steps were further predicted using molecular docking. This study not only clarified that gigantol was one of the biosynthetic intermediates of erianin, but also determined the biosynthetic pathway from gigantol to erianin, and identified hydroxylases and OMTs as the key modifying enzymes during the steps of this pathway. These findings would provide a clearer understanding of the biosynthetic pathway of erianin, and more attention should be devoted to the investigation on the biosynthetic pathway from 3,3’5-trihydrobibenzyl to gigantol and related enzymes.

## Data Availability

The datasets presented in this study can be found in online repositories. The names of the repository/repositories and accession number(s) can be found in the article/**Supplementary Material**.
