# Supplementary material for: Integrated metabolomic and transcriptomic analyses of Dendrobium chrysotoxum and D. thyrsiflorum reveal the biosynthetic pathway from gigantol to erianin
Source: Front Plant Sci. 2024 Sep 26;15:1436560. doi: 10.3389/fpls.2024.1436560 (PMC11464314; doi:10.3389/fpls.2024.1436560)
Supplement: Supplementary file 1 [file DataSheet1.docx]

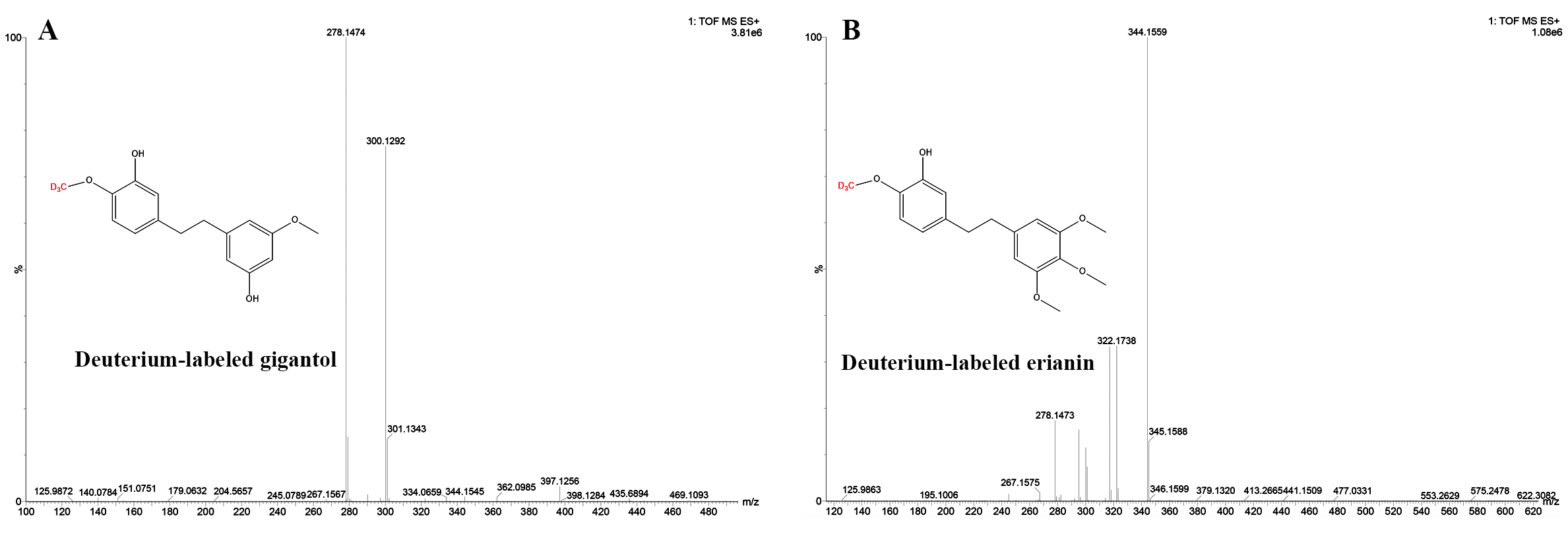


**Fig. S1.** The MS/MS spectra of standards deuterium-labeled gigantol at 5.98 min (**A**) and erianin at 6.17 min (**B**).


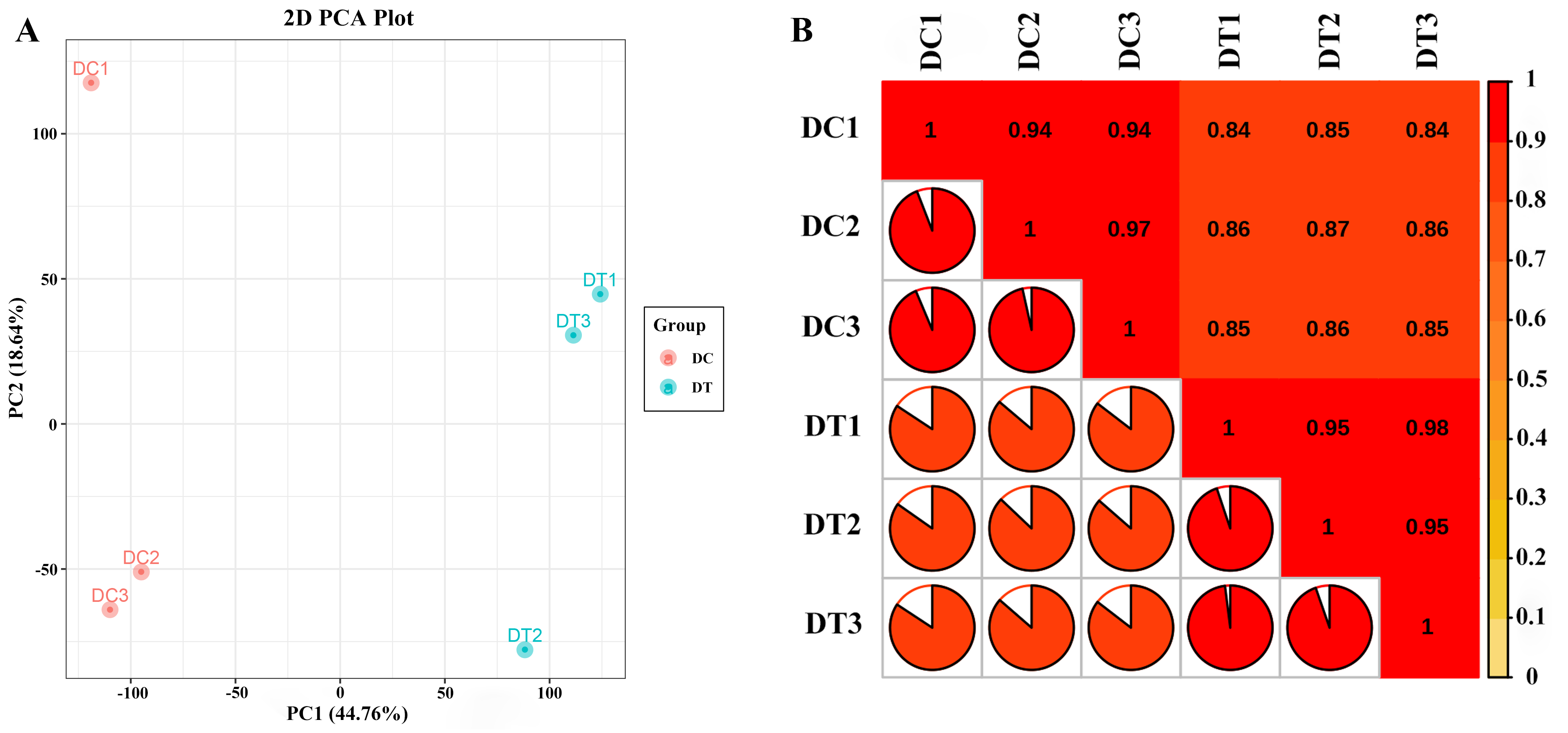


**Fig. S2.** Principal component analysis (PCA) (**A**) and correlation analysis (**B**) of transcriptome data between DC and DT.
